# Spectral quantitative and semi-quantitative EEG provide complementary information on the life-long effects of early childhood malnutrition on cognitive decline

**DOI:** 10.3389/fnins.2023.1149102

**Published:** 2023-09-15

**Authors:** Fuleah A. Razzaq, Ana Calzada-Reyes, Qin Tang, Yanbo Guo, Arielle G. Rabinowitz, Jorge Bosch-Bayard, Lidice Galan-Garcia, Trinidad Virues-Alba, Carlos Suarez-Murias, Ileana Miranda, Usama Riaz, Vivian Bernardo Lagomasino, Cyralene Bryce, Simon G. Anderson, Janina R. Galler, Maria L. Bringas-Vega, Pedro A. Valdes-Sosa

**Affiliations:** ^1^The Clinical Hospital of Chengdu Brain Science Institute, MOE Key Lab for Neuroinformatics, University of Electronic Science and Technology of China, Chengdu, China; ^2^Cuban Neuroscience Center, La Habana, Cuba; ^3^Montreal Neurological Institute, McGill University, Montreal, QC, Canada; ^4^Faculty of Psychology, Universidad Autónoma de Madrid, Madrid, Spain; ^5^National Center for Animal and Plant Health, CENSA, San José de las Lajas, Mayabeque, Cuba; ^6^Facultad de Psicologia, Universidad La Habana, La Habana, Cuba; ^7^The George Alleyne Chronic Disease Research Centre, Caribbean Institute for Health Research, University of the West Indies, Cave Hill, Barbados; ^8^The George Alleyne Chronic Disease Research Centre, Caribbean Institute for Health Research, University of the West Indies, Cave Hill, Barbados; ^9^Division of Pediatric Gastroenterology and Nutrition, MassGeneral Hospital for Children, Boston, MA, United States

**Keywords:** malnutrition, EEG, qEEG, grand total EEG, latent variable, item response theory, cognitive decline

## Abstract

**Objective:**

This study compares the complementary information from semi-quantitative EEG (sqEEG) and spectral quantitative EEG (spectral-qEEG) to detect the life-long effects of early childhood malnutrition on the brain.

**Methods:**

Resting-state EEGs (*N* = 202) from the Barbados Nutrition Study (BNS) were used to examine the effects of protein-energy malnutrition (PEM) on childhood and middle adulthood outcomes. sqEEG analysis was performed on Grand Total EEG (GTE) protocol, and a single latent variable, the semi-quantitative Neurophysiological State (sqNPS) was extracted. A univariate linear mixed-effects (LME) model tested the dependence of sqNPS and nutritional group. sqEEG was compared with scores on the Montreal Cognitive Assessment (MoCA). Stable sparse classifiers (SSC) also measured the predictive power of sqEEG, spectral-qEEG, and a combination of both. Multivariate LME was applied to assess each EEG modality separately and combined under longitudinal settings.

**Results:**

The univariate LME showed highly significant differences between previously malnourished and control groups (*p* < 0.001); age (*p* = 0.01) was also significant, with no interaction between group and age detected. Childhood sqNPS (*p* = 0.02) and adulthood sqNPS (*p* = 0.003) predicted MoCA scores in adulthood. The SSC demonstrated that spectral-qEEG combined with sqEEG had the highest predictive power (mean AUC 0.92 ± 0.005). Finally, multivariate LME showed that the combined spectral-qEEG+sqEEG models had the highest log-likelihood (−479.7).

**Conclusion:**

This research has extended our prior work with spectral-qEEG and the long-term impact of early childhood malnutrition on the brain. Our findings showed that sqNPS was significantly linked to accelerated cognitive aging at 45–51 years of age. While sqNPS and spectral-qEEG produced comparable results, our study indicated that combining sqNPS and spectral-qEEG yielded better performance than either method alone, suggesting that a multimodal approach could be advantageous for future investigations.

**Significance:**

Based on our findings, a semi-quantitative approach utilizing GTE could be a valuable diagnostic tool for detecting the lasting impacts of childhood malnutrition. Notably, sqEEG has not been previously explored or reported as a biomarker for assessing the longitudinal effects of malnutrition. Furthermore, our observations suggest that sqEEG offers unique features and information not captured by spectral quantitative EEG analysis and could lead to its improvement.

## Highlights

– Participants with protein-energy malnutrition (PEM) limited to the first year of life have significantly more EEG abnormalities than controls (CON) in childhood and middle adulthood. These differences can be reliably determined using GTE items from visual inspection of the EEG.
– Increased sqEEG abnormalities in childhood and adulthood predicted accelerated cognitive decline up to 40 years later in middle adulthood.
– A multimodal approach combining spectral-qEEG and sqEEG yields the most accurate results distinguishing PEM from controls.
– sqEEG contributes additional information about PEM effects on EEG, which spectral-qEEG does not capture.

## 1. Introduction

Recent research has demonstrated that exposure to adverse childhood experiences during critical developmental phases can negatively impact both physical and mental health (Hawkins et al., 2019; Short and Baram, 2019; Bing-Canar et al., 2022; Tsotsoros et al., 2022; Rokach and Clayton, 2023). Childhood malnutrition, which accounts for nearly half of all deaths of children under 5^1^ is a prevalent and significant form of childhood adversity. Despite improvements in public health measures and survival rates, the long-term effects of childhood malnutrition on brain development throughout an individual’s lifespan remain concerning. There are currently few if any scalable and cost-effective measures for identifying brain dysfunction due to this condition (Galler et al., 2021). Therefore, there is a pressing need to develop brain imaging techniques that can directly assess the neurodevelopmental consequences of malnutrition, especially in low resource settings.

Our research group has addressed this issue by examining the use of the Electroencephalogram (EEG) as a viable technology to investigate the neurodevelopmental consequences of protein-energy malnutrition (PEM) in the first year of life (Taboada-Crispi et al., 2018; Bringas Vega et al., 2019; Bosch-Bayard et al., 2022). This work has leveraged the Barbados Nutrition Study (BNS), a longitudinal study spanning over 50 years, aimed at investigating the effects of protein-energy malnutrition (PEM) aimed at investigating the effects of early PEM over the life span (Galler et al., 1983, 2012). Study participants had normal birth weights but experienced moderate-severe protein-energy malnutrition limited to the first year of life. BNS subjects were identified as infants, and enrolled in a national intervention program (see Methods) that followed the children until 12 years of age, ensuring no further exposure to malnutrition. The control group were classmates who were selected in childhood and matched to the affected individuals by age, sex, and handedness. EEG was collected on subjects from these cohorts at ages 5–11 years (school age) and 45–51 years (middle adulthood).

Before summarizing our prior EEG results for these BNS cohorts, it is necessary to note that two different modalities can extract potential EEG biomarkers:

- Spectral quantitative EEG (spectral-qEEG) quantifies the EEG through the power spectrum of activity at the channels or the sources. Our group has contributed to developing this technique for several decades (John et al., 1977; Valdés et al., 1992; Bosch-Bayard et al., 2001; Li et al., 2022) and demonstrated its accuracy for detecting brain abnormalities and subject classification. Spectral-qEEG is objective and easy to deploy, the significant stumbling block being the need to eliminate the effect of EEG artifacts.
- Semi-quantitative EEG (sqEEG) involves scores by expert subjective evaluations of EEG with standardized ratings that are later fully quantified employing statistical techniques. Such scoring of expert evaluation is a common technique in psychometrics (e.g., Likert scale) and was pioneered in imaging (Scheltens et al., 1993). The usefulness of sqEEG is also an established technique in clinical neuroscience (Pijnenburg et al., 2008; Roks et al., 2008; Micanovic and Pal, 2014; Barcelon et al., 2019). The disadvantage of this technique is the need for expert evaluation which is a limiting factor for its diffusion.

A current and trenchant discussion in clinical neurophysiology com compares the relative merits of spectral-qEEG and sqEEG. As the existence of automatic EEG measures, such as spectral-qEEG, might supplant sqEEG. However, this is not the case. While spectral-qEEG is considered an essential complement to visual inspection of the EEG, visual inspection of the EEG is still considered the gold standard in clinical neurophysiology and is constantly being revised (Niedermeyer and Lopes da Silva, 2005; Beniczky et al., 2017; Wüstenhagen et al., 2022). Efforts are being made to compare the results of this visual inspection with spectral-qEEG, a goal pursued by several International Federation of Clinical Neurophysiology (IFCN)^2^ and the Global Brain Consortium Project (GBC)^3^ (*Global Brain Consortium Project: Global EEG Norms*).

One of the hypotheses underlying this paper is that sqEEG reflects, in addition, to rough background frequency information, additional features in the “grapho-elements” that arise from nonstationary and nonlinear harmonic signal relations, to which spectral analysis is blind. Rather than contraposing spectral-qEEG and sqEEG, we believe it essential to document differences between the two EEG modalities, thus enabling the discovery of additional features needed to improve spectral-qEEG. Beyond adding multivariate cross-spectral EEG information to qEEG, as in (Li et al., 2022), an arsenal of nonlinear EEG measures (Stam, 2005) is already available to be evaluated against visual inspection. Our earlier studies only gave partial answers in terms of comparing these two EEG modalities.

We previously reported that spectral-qEEG has provided biomarkers that stably and accurately classified between PEM and control groups at 5–11 years of age with an area under the ROC curve (AUC) of 0.83 and 0.82 for the scalp (Taboada-crispi et al., 2018) and source spectra (Bringas Vega et al., 2019), respectively. sqEEG achieved an AUC of 0.83 (for the best-performing expert). AUC was thus statistically indistinguishable for spectral-qEEG and sqEEG according to their sampling distribution. What was not addressed in the cited studies, nor to our knowledge in the literature, was whether spectral-qEEG and sqEEG precision accuracy arises from similar or distinct sources of information about the EEG. Note that these studies only evaluated classification at school age. Another study by our group assessed the life-long trajectory spectral-qEEG measures for the BNS cohort and showed significant differences in source spectra between the PEM/CON groups and between school-age/adulthood (Bosch-Bayard et al., 2022). These changes were significantly associated with accelerated cognitive decline in the PEM group.

The current study aims to address the failure to systematically compare spectral-qEEG and sqEEG measures of early malnutrition throughout the lifespan. To address this research gap, we contrast changes in both EEG modalities (spectral-qEEG as a function of nutrition status and age). Importantly, we validate these differences by comparing them with cognitive outcomes in adulthood as measured by the Montreal Cognitive Assessment (MoCA), a well-established scale for measuring mild cognitive impairment (Nasreddine et al., 2005). We also compare the classification accuracy of both EEG modalities across the life span at 5–11 years and 45–51 years. An important design consideration of our analysis is to identify whether both EEG modalities convey the same information regarding the brain effects of malnutrition or reflect different aspects of the EEG signal.

## 2. Materials and methods

**Figure 1:**
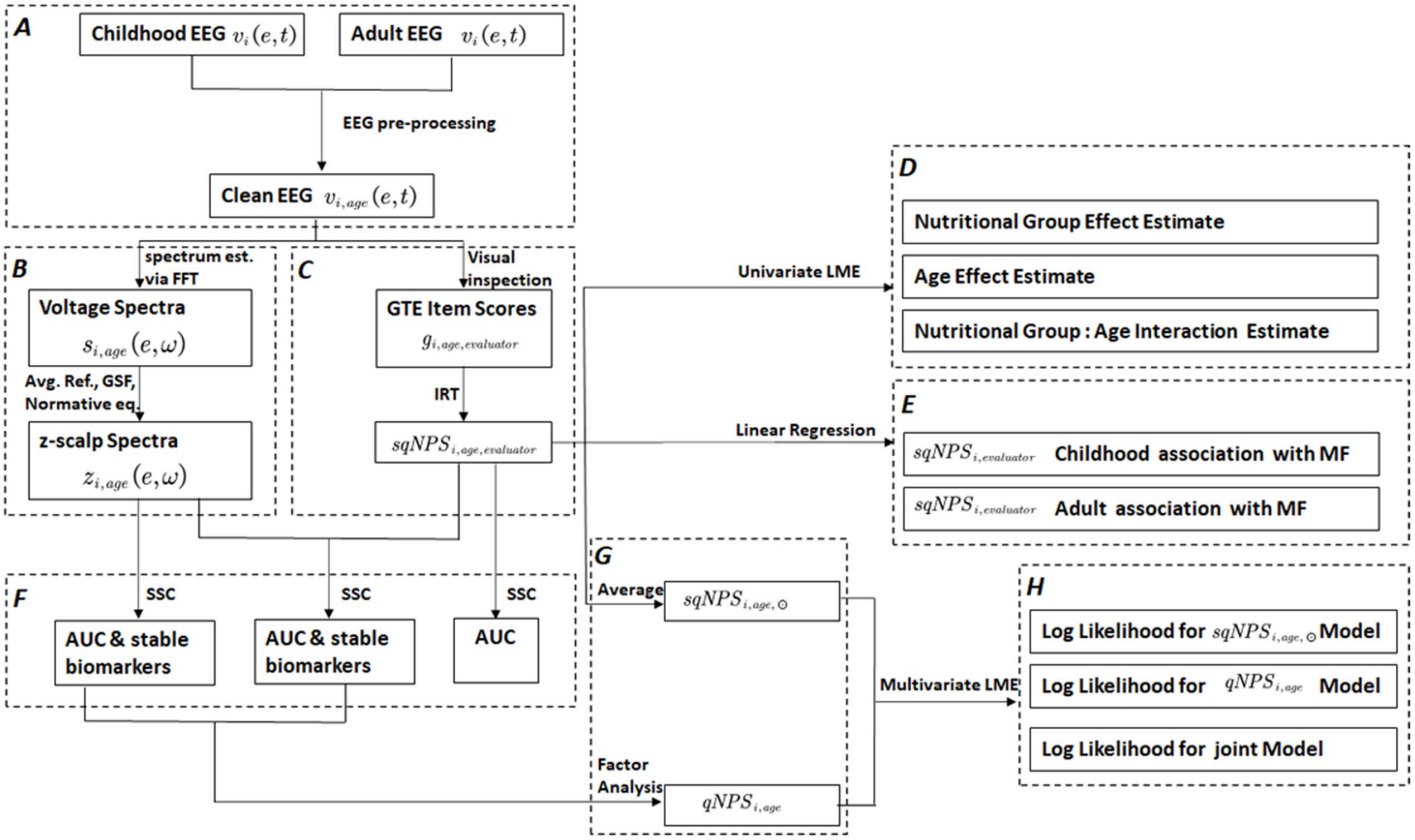
This flowchart outlines the various processing steps and outputs involved in the study. The edges represent the applied processes, while the nodes/rectangles depict the outcomes of different processing steps. The dashed rectangles indicate a sub-process with multiple steps. These include **(A)** preprocessing of raw EEG $v_{i,\text{age}}\left(e,t\right)$, **(B)** processing pipeline for spectral quantitative EEG (spectral-qEEG) z-scalp spectra $z_{i,\text{age}}\left(e,\omega \right)$ using Fast Fourier Transform (FFT), average reference, Global Scale Factor (GSF), and normative equations, **(C)** processing steps for semi-quantitative Neurophysiological status (${\text{sqNPS}}_{i,\text{age},\text{evaluator}}$): visual inspection to generate Grand Total EEG (GTE) scores $G_{i,\text{age},\text{evaluator}}$ and Item Response Theory (IRT) based latent factor, **(D)** univariate linear mixed effect (LME) modal analysis: to estimate the effects of nutritional group, age, and group: age interaction on ${\text{sqNPS}}_{i,\text{age},\text{evaluator}}$. **(E)** Validation of ${\text{sqNPS}}_{i,\text{age},\text{evaluator}}$ scores based on MoCA Factor ($MF_{i,\text{age}=45-51\text{yrs}}$), **(F)** application of the stable sparse classifier (SSC) for selecting stable qEEG biomarkers and comparing the prediction accuracy of $z_{i,\text{age}}\left(e,\omega \right)$ and ${\text{sqNPS}}_{i,\text{age},\text{evaluator}}$ separately and combined, **(G)** transformation into a single dimension by applying factor analysis to qEEG, resulting in quantitative Neurophysiological status (${\text{qNPS}}_{i,\text{age}}$), and averaging ${\text{sqNPS}}_{i,\text{age},\text{evaluator}}$ over evaluators to produce Average sqNPS (${\text{sqNPS}}_{i,\text{age},⊙}$) scores, **(H)** multivariate LME for longitudinal model comparisons for ${\text{qNPS}}_{i,\text{age}}$ and ${\text{sqNPS}}_{i,\text{age},⊙}$ separately and combined.

In this paper, we employ different symbols and acronyms, and their explanations are available in Table 1 to ensure clarity and ease of comprehension. A flowchart in Figure 1 also provides an overview of the methods and procedures.

**Table 1:** Symbols used in the paper and their explanation.

Symbol	Acronyms	Description
–	PEM	Protein Energy Malnutrition
–	CON	Controls
i	–	$i^{th}$ Subject
age	–	Childhood or Adult age to represent two studies
$v_{i,\text{age}}\left(e,t\right)$	Time-domain EEG	EEG recordings for the electrode e at the time t
$s_{i,\text{age}}\left(e,\omega \right)$	voltage spectra	Voltage spectra at electrode e and frequency ω
$z_{i,\text{age}}\left(e,\omega \right)$	z-scalp spectra	Quantitative EEG for the electrode e at the frequency ω
$G_{i,\text{age},\text{evaluator}}$	GTE Scores	Grand Total EEG (GTE) scores
${\text{sqNPS}}_{i,\text{age},\text{evaluator}}$	semi-quantitative (sq) Neurophysiological status	Latent sqEEG factor generated from item Response theory (IRT) based on GTE scores
${\text{sqNPS}}_{i,\text{age},⊙}$	Average sqNPS	Averaged over multiple evaluators for sqNPS
${\text{qNPS}}_{i,\text{age}}$	quantitative (q) Neurophysiological status	Latent spectral-qEEG variable generated via factor analysis of spectral-qEEG biomarkers selected by the stable sparse classifier (SSC)
$MF_{i,\text{age}=45-51\text{yrs}}$	MoCA Factor	IRT-based factor generated from Montreal Cognitive Assessment (MoCA) sub-scores

### 2.1. Study site

The current study was conducted in Barbados, a Caribbean country whose population was 281,635 in 2022. The demographic makeup is 92% African/Caribbean origin, 4% Caucasian, and 4% individuals of Asian, Lebanese, and Syrian descent. In 1970, when the study began, the infant mortality rate was 46 per 1,000 live births. Today it stands at 9.3, and Barbados was ranked 70/193 countries and territories (and classified as a High level of human development) on the Human Development Index in 2021-2022 (Conceição et al., 2022). Malnourished participants (N = 129, 52 females, 77 males) were born between 1967 to 1972. They were clinically diagnosed with Grade II-III protein-energy malnutrition (PEM, weight below 75% of expected weight for age, in the absence of edema) limited to the first year of life. Selection criteria were as follows: birth weight ≥ 2,500 grams; APGAR scores ≥8, no pre or perinatal complications; and no childhood encephalopathic events (Galler et al., 1983). Controls (CON, *N* = 129, 52 females, 77 males) were healthy classmates of the PEM children matched by age, sex, and handedness who met the same inclusion criteria but had no histories of malnutrition. After discharge from the hospital, all PEM children were enrolled in a government-supported intervention program from ages 0–12 years, which provided food subsidies, nutrition education for the primary caretaker, a 2–3 day/week nursery program, routine medical care, and regular home visits (Ramsey, 1979; Ramsey et al., 1986). These participants have been comprehensively evaluated over seven waves of data collection between 1977 and 2018.

### 2.2. Research design

EEGs were first recorded in this sample in 1977–1978 (*n* = 258) when the subjects were between 5 and 11 years of age. However, only 108 original EEG recordings (CON = 62, PEM = 46) were available during follow-up (Taboada-Crispi et al., 2018). In 2018, we conducted a 40-year follow-up study of a subset of 100 participants from the original cohort, and EEGs were again recorded at this time point (Bosch-Bayard et al., 2022). Ninety-four individuals had valid EEGs at 45–51 years (CON = 51, PEM = 43). Thus, 202 valid EEG recordings were available for the current study, and 53 participants had EEG data available in both childhood and adulthood. For more details about the BNS longitudinal study design and the flow of the samples (see Figure 1; Bosch-Bayard et al., 2022).

Note that the reduction in sample size between 1978 and 2018 was primarily due to funding constraints, requiring us to limit this later study phase to 100 participants. Furthermore, we were unable to locate some of the original participants for whom 1977 EEG data were valid for the following reasons: some participants lived overseas, others did not respond to requests for contact or refused to participate, and some were deceased, in prison, and/or severely ill. We have previously reported that the 1978 childhood characteristics of the participants vs. non-participants in the 2018 study were similar (see Table 1A, Bosch-Bayard et al., 2022). To confirm if the missing cases affected the results presented here, we implemented a sensitivity analysis for the ignorability assumption of data missingness; details are in the statistical analysis section below.

Informed consent was provided by the parents of all participants when they were initially enrolled in the study under Protocol E1962, approved by the Boston University Medical Center Institutional Review Board and the Barbados Ministry of Health Ethics Committee. Current oversight was provided by the Massachusetts General Brigham IRB (Protocol No. 2015P000329, approved to 1/19/25), the Cuban Neurosciences Center (2017/02/17/CNEURO), and the Barbados Ministry of Health and Wellness. All adult participants provided written informed consent and were compensated for their time and travel expenses.

### 2.3. Measures

#### 2.3.1. EEG recording

The original 1978 EEG recordings were obtained using the DEDDAS EEG equipment (sampling rate 100 Hz). The protocol has been described elsewhere in detail (Taboada-crispi et al., 2018). For the 2018 follow-up study, EEG recordings were performed using the same procedures as those in the earlier study using the MEDICID system at a sampling rate of 200 Hz (Neuronic, Havana, Cuba). The equivalence of both recording systems is described in Appendix 1, Bosch-Bayard et al. (2022). We used standard protocols developed jointly by the Brain Research Laboratories, NYU, and the Cuban Neuroscience Center (Hernandez-Gonzalez et al., 2011), using IFCN guidelines for resting-state EEG analysis (International Federation of Clinical Neurophysiology IFCN; see footnote 1). The EEG acquisition protocol for both studies was as follows:

Data was collected and visually inspected by two neurophysiologists, who were blind to the subjects’ malnutrition history. Nineteen surface electrodes were placed (Fp1, Fp2, Fz, F3, F4, F7, F8, Cz, C3, C4, T3, T4, T5, T6, Pz, P3, P4, O1, and O2) according to the international 10–20 system, referenced to linked earlobes. The electrode impedance was kept below 5 kΩ. Subjects were seated on a comfortable reclining armchair in a quiet, air-conditioned room and were instructed to stay awake. Resting-state EEGs were recorded for 8–10 min. Note that in 1978 only eye-closed EEGs were collected; in 2018, the EEG was recorded under different conditions (open and closed eyes, hyperventilation, and recovery). Subjects were monitored regularly by the neurophysiologist for their wakefulness. Throughout the recording, subjects were also asked to report if they were awake.

The raw EEG data is denoted as $v_{i}\left(e,t\right)$, here i is, the subject, e and t are EEG electrode and time, respectively. The $v_{i}\left(e,t\right)$ processing and cleaning method was:

- For both sqEEG and spectral qEEG, visual inspection was used to remove bad channels, interference by power lines interference, EKG, EMG. teeth grinding, eye or body movement, and high electrode impedance.
- Spectral qEEG artifact rejection was supplemented by independent component analysis (ICA)and the AAR plug-in from the EEGLAB 13.6.5b toolbox (Gomez-Herrero et al., 2006). Additionally, the neurophysiologists conducted visual inspections to select at least 1 min of EEG epochs free of artifacts that lasted 2.56 s.

EEG Pre-processing resulted in clean data (Figure 1A) denoted as $v_{i,\text{age}}\left(e,t\right)$, here, age represents the childhood or adult age to accommodate two studies.

#### 2.3.2. Grand total EEG scale

Two clinical neurophysiologists from the Cuban Neuroscience Center evaluated the $v_{i,\text{age}}\left(e,t\right)$ independently for both studies (Figure 1C). They were blind to the participant’s malnutrition history. A modified version of the GTE scale was implemented (Jonkman, 1989; de Weerd and Perquin, 1990; Claus et al., 1999; Marcuse et al., 2016) denoted here as $G_{i,\text{age},\text{evaluator}}$ which was composed of 6 items. Here i is the subject, age is childhood or adult age and evaluator represents the neurophysiologist who performed the visual inspection. $G_{i,\text{age},\text{evaluator}}$ items were graded between 0 and 5 (Kane et al., 2017).

1. Frequency of rhythmic background activity (the predominant EEG activity observed in resting state. This activity is commonly associated with alpha activity, although this may vary depending on the recording conditions)
  0= > 8 Hz, 1 = 7-8 Hz, 2 = 6-7 Hz
2. Diffuse slow activity (the occurrence of persistent non-rhythmic delta and slow theta waves localized in broader regions)
  0 = None, 1 = Slow theta, 2 = Intermittent theta + sporadic delta, 3 = Intermittent theta + intermittent delta
3. Reactivity of rhythmic background activity
  0 = Normal, 1 = Decreased with eye-opening, 2 = Absent with eye-opening
4. Focal abnormality (the occurrence of EEG abnormality that is localized topographically)
  0 = No focal abnormality, 1 = Slight unilateral abnormality, 2 = Slight bilateral abnormality, 3 = Severe unilateral + Slight contralateral, 4 = Severe bilateral, 5 = Multifocal
5. Paroxysmal activity (the occurrence of activity that has a sudden rapid onset, rapid attainment of a maximum, abrupt termination, and is distinguished from background activity. Such as spikes and spike-and-waves. Spikes have a duration between 20 and 70 msec by convention)
  0 = None, 1 = Paroxysmal slow activity, 2 = Spikes, 3 = Spike and wave (one Spike followed by a delta frequency wave)
6. Sharp wave activity (Paroxysmal activity that has a lower initial slope than spikes with a duration between 70-and 200 ms).
  0 = None, 1 = Sporadic sharp waves, 2 = Frequent sharp waves.

For the 1978 analyses, only five GTE items were used since the “Reactivity of rhythmic background activity” must be evaluated by comparing the resting state to other conditions which were not included in this earlier study.

#### 2.3.3. Montreal cognitive assessment

The Montreal cognitive assessment (MoCA) was administered to participants in middle adulthood (at 45–51 years) by a researcher blind to the participant’s nutrition group. The MoCA comprises seven subscales, assessing visuospatial and executive functions, naming, attention, language, abstraction, memory, and orientation. It is a validated screening measure for mild cognitive impairment (MCI) (Nasreddine et al., 2005). The highest possible score is 30, and the threshold below which MCI is suspected is 26.

#### 2.3.4. Covariates

Childhood standard of living was measured at three time points in childhood and adolescence using the 50-item Barbados Ecology Questionnaire (Galler and Ramsey, 1985; Galler et al., 2012), which assessed conditions in the home, parental educational achievement, and type of employment. Factor analysis, based on data combined across all three-time points, identified a first principal component that appeared to represent the household standard of living, including the presence of a refrigerator, closet, bathroom, television, electricity, running water, and gas or electric cooking fuel; the number of bedrooms/rooms; household food expenditure; type of toilet; and weekly household income. Scores based on this factor were standardized to have zero mean and unit variance. The Hollingshead Scale (Hollingshead and Redlich, 1963) was used to evaluate the adult participants’ educational level and employment status to measure adult SES (socioeconomic status).

### 2.4. Statistical analyses

#### 2.4.1. Semi-quantitative EEG

Using Item Response Theory (IRT), we obtained a latent variable underlying all GTE items for sqEEG. This approach is used as the indicator variables were categorical and not continuous. A polytomous IRT was implemented as the responses ranged between 0 and 5 (Beaujean, 2014; Chalmers, 2015). The analysis was conducted via R package MIRT (Chalmers, 2012), utilizing a generalized partial credit model (Pollitt and Hutchinson, 1987; Chalmers, 2015). The latent variable, “semi-quantitative Neurophysiological status” (sqNPS), was created based on the most informative items with the highest loadings. Therefore, the final latent score was the optimal combination of measured items based on their information content. Notably, the high factor loadings were solely focused on items with a clear separation of probabilities between the different levels of the item scale and not between the two groups (CON vs. PEM), as responses for all participants were included in the IRT analysis. Thus the construction of the latent factor did not bias subsequent analysis. The ${\text{sqNPS}}_{i,\text{age},\text{evaluator}}$ was estimated using the original raw item scores from five common GTE items for the 1978 and 2018 datasets. Here i is the subject, age is childhood or adult age to represent the two time points of data collection and evaluator represents the neurophysiologist who performed the visual inspection (Figure 1C). When we needed to pool the results of both evaluators, we used the average of the two ${\text{sqNPS}}_{i,\text{age},\text{evaluator}}$ denoted as ${\text{sqNPS}}_{i,\text{age},⊙}$ (Figure 1G). Considering individual subjects, the sample size was *N* = 202 (108 in 1978 and 94 in 2018). However, the number of repeated measures doubled when judging the effect of differences between evaluators. We assessed alternative IRT factor models based on item loadings and trace plots and optimized them using model fit indices such as Akaike’s Information Criterion (AIC) and the Bayesian Information Criterion (BIC).

#### 2.4.2. Spectral quantitative EEG

We processed the EEG recordings $v_{i,\text{age}}\left(e,t\right)$ using the methods outlined in Bosch-Bayard et al. (2022); EEGs were re-referenced to the Average Reference. To convert the time-domain scalp EEG signals to the frequency domain, we used the Fast Fourier Transform (FFT). We then calculated spectral matrices $s_{i,\text{age}}\left(e,\omega \right)$ using 24 EEG epochs for most cases, with a minimum of 20 to match both studies. These calculations produced a spectral matrix with 48 frequency bins ranging from 0.78 Hz to 19.15 Hz denoted as ω. As reported by Bosch-Bayard et al. (2022), we compared the technical parameters for both systems (1978 and 2018). We found them practically equivalent with no significant bias. To correct the EEG spectra at the scalp and eliminate scale variability in the EEG signal that is not related to physiological sources, we used the Global Scale Factor (GSF). This step is beneficial when comparing recordings obtained from different hardware. More details about this can be found in our previously published study (Bosch-Bayard et al., 2022).

Furthermore, normative EEG norms (Bosch-Bayard et al., 2020) were applied to the log spectra. This process produced z scalp spectra (Figure 1B) for each channel and frequency $z_{i,\text{age}}\left(e,\omega \right)$. Here e is the EEG electrode or channel, and ω is the frequency. The z-scalp spectra $z_{i,\text{age}}\left(e,\omega \right)$ were screened for stable biomarkers, then used to construct a univariate latent variable ${\text{qNPS}}_{i,\text{age}}$ via factor analysis (Figure 1G). The details about these steps can be found in subsections 2.4.5–2.4.6.

#### 2.4.3. Univariate linear mixed effect model for longitudinal sqNPS

We incorporated the latent variable ${\text{sqNPS}}_{i,\text{age},\text{evaluator}}$ into a linear mixed effect (LME) model (Figure 1D) to examine the differences in longitudinal evolution between nutrition groups. LME models are statistical models that can analyze data with repeated measures, accounting for the random variations between subjects or evaluator not explained solely by the fixed effects, thus leading to more precise estimates of the effects of interest. To implement mixed-effect modeling, we utilized the *lme* function from the *nlme* R package (Pinheiro et al., 2020). We modeled age (in years) and nutrition group as the main/fixed effects, with an interaction term between age and nutrition group to estimate any differences in slope over the life span. Sex and standard of living (Ecology Factor) were also modeled as covariates, and their effects were adjusted. Participants were modeled as random effects to account for the repeated measures. Furthermore, we implemented another model where the participants and evaluator were included as random effects to eliminate any evaluation biases. We compared both models via R ANOVA function to select the optimal model.

##### 2.4.3.1. Sensitivity analysis for missing data

As mentioned before, when describing data collection, EEG recordings in 2018 were limited by funding constraints, producing a missing data issue. In order to assess the validity of the ignorability assumption, we conducted a sensitivity analysis. The ignorability assumption is based on the notion that all confounding factors should be adjusted-for to infer from the regression coefficient as average effects. This assumption makes the treatment assignment ignorable, like a completely randomized experiment. However, the missing data mechanisms, such as Missing Not at Random (MNAR), can violate this assumption. To evaluate the effects of missing data mechanisms on the LME model estimates, we used the R package ISNI (Ma et al., 2005; Xie et al., 2018) to apply an index of local sensitivity to non-ignorability (ISNI). ISNI provides a standardized sensitivity index “c” where a large c indicates that LME estimates are robust, and only extreme violations of the ignorability assumption can alter the initial estimates. Therefore, non-ignorability is of little concern. According to a guideline proposed by Ma et al. (2005), a rule of thumb is that c > = 1 demonstrates robust estimates.

#### 2.4.4. Association of *sq*NPS with cognitive outcomes

To validate the ${\text{sqNPS}}_{i,\text{age},\text{evaluator}}$ with the Montreal Cognitive Assessment (MoCA), used for detecting mild cognitive impairment (MCI), we compared MOCA scores with ${\text{sqNPS}}_{i,\text{age},\text{evaluator}}$ scores (Figure 1E). The MOCA test assesses visuospatial and executive functions through its seven subscales: naming, attention, language, abstraction, memory, and orientation. To reduce the dimensionality of the MOCA variables, we applied polytomous IRT and obtained a latent MOCA factor ($MF_{i,\text{age}=45-51\text{yrs}}$). We applied two linear regression analysis to determine the relationship between (i) ${\text{sqNPS}}_{i,\text{age}=5-11\text{yrs},\text{evaluator}}$ and $MF_{i,\text{age}=45-51\text{yrs}}$ (ii) ${\text{sqNPS}}_{i,\text{age}=45-51\text{yrs},\text{evaluator}}$ and $MF_{i,\text{age}=45-51\text{yrs}}$, while adjusting for age, sex, and standard of living.

#### 2.4.5. Discriminant analysis for prediction power

In order to evaluate the effectiveness of ${\text{sqNPS}}_{i,\text{age},\text{evaluator}}$ and how it compares to $z_{i,\text{age}}\left(e,\omega \right)$ as a longitudinal biomarker for malnutrition, we applied a stable sparse classifier (SSC) (Bosch-Bayard et al., 2018), as shown in Figure 1F. This method divides samples into training (70%) and test sets to avoid random selection. SSC implements the “independent significant features (IndFeat)” procedure to screen variables, and the elastic net regression method selects the best variables for classification. This process is repeated 1,000 times, and only significant features in over 50% of the iterations are kept for further analysis. Receiver operator curves(ROCs) are generated to evaluate the expected classification accuracy, carried out on an independent set of 1,000 cross-validations. The median ROC is used to summarize the classifier’s operational characteristics. Furthermore, the distribution of the 1,000 areas under the ROC curve (AUC) is used to fit a kernel probability density that quantifies the variability of classification performance with variable selection.

#### 2.4.6. Multivariate linear mixed effect model

Screened variables from the stable sparse classifier (SSC) were employed as stable biomarkers to test for the nutritional group differences. The selected variables for $z_{i,\text{age}}\left(e,\omega \right)$ were transformed via factor analysis for dimensionality reduction, creating a new latent variable named quantitative NPS (${\text{qNPS}}_{i,\text{age}}$).

These new variables were utilized to implement multivariate LME models (Figure 1H). The idea is to implement linear mixed effect models (multilevel modeling) to evaluate both modalities under longitudinal settings and repeated measure design. A third model was also implemented for a joint estimate. We implemented these models under multivariate design, where the model’s outcome becomes a matrix with two columns $\left[{\text{qNPS}}_{i,\text{age}},{\text{sqNPS}}_{i,\text{age},⊙}\right]$. The Multivariate linear mixed effect (MLME) estimated the combined effect of malnutrition on both dependent variables.

Furthermore, we created two other models from the main MLME model. These models were implemented such that coefficients related to one of the modalities were forced to be zero. We have implemented these models via MLME to make these models comparable in terms of model fit indices and to evaluate the optimal biomarkers under longitudinal settings.

R ANOVA function was applied for model comparison. The ANOVA function is a statistical tool used to compare multiple models and determine if the difference in fit between them is significant. The ANOVA functions work with log-likelihood values and Log Likelihood ratio (L ratio). The log-likelihood is the probability of observing the data given the model parameters and measures how well the model fits the data. The higher the log-likelihood, the better the model fits the data.

On the other hand, the L ratio measures the improvement in model fit when comparing two nested models. It is calculated as the difference in log-likelihoods between the two models. Moreover, the Log-Likelihood of the different models is compared using a chi-squared test to determine if the difference is statistically significant. If the value of p of the chi-squared test is less than 0.05, in that case, we reject the null hypothesis that the models are equally good fits and conclude that one model is a better fit than the other based on the log-likelihood values.

## 3. Results

### 3.1. Demographics

Table 2 displays the demographic characteristics of the study participants. The table confirms that the PEM and CON groups did not differ in age, sex, or handedness. However, PEM participants were disadvantaged in childhood with respect to their household standard of living (Childhood Ecology Factor), consistent with earlier publications in this cohort (Galler et al., 1983). The standard of living is therefore adjusted in all analyses reported below.

**Table 2:** Childhood demographic characteristics mean and standard deviation of protein energy malnourished (PEM) and control (CON) participants.

	PEM	CON	*t* or χ^2^	Value of *p*
*N* (%)	66 (44.3)	83 (55.7)		
Males (%)	38 (57.6)	50 (60.2)	0.11	0.72
Age in 1977 (years)	8.10 ± 1.83	7.85 ± 1.89	0.81	0.418
Age in 2018 (years)	48.77 ± 1.83	48.52 ± 1.89	0.81	0.418
Left-handedness (%)	4 (6.1)	3 (3.6)	0.49	0.483
Childhood standard of living (5–11 years)	−1.056 ± 0.836	0.189 ± 0.896	−6.11	<0.0001

### 3.2. GTE items

Visual inspection of the EEG recordings using the GTE items demonstrated a higher rate of abnormalities in the PEM group compared to the controls in both the 1978 and 2018 datasets, with more abnormal findings reported at the older ages in 2018 than in children (1978). The total abnormalities found at ages 5–11 years have been previously reported (Taboada-Crispi et al., 2018). At ages 45–51 years, the total number of EEG recordings with abnormalities was 63/94, with 35 (77.7%) from the PEM group and 28 (52.7%) from the Control group (χ^2^ with Yates correction = 5.79, *p* = 0.016).

### 3.3. sqEEG: sqNPS score

In this study, we initially employed an Item Response Theory (IRT) model with five items to investigate the relationship between the GTE (Grand Total EEG) items and the latent factor ${\text{sqNPS}}_{i,\text{age},\text{evaluator}}$(Neurophysiological status). The results revealed that the “Background Frequency” item had a low loading of 0.134 and was not associated with the ${\text{sqNPS}}_{i,\text{age},\text{evaluator}}$. Additionally, we observed that the “Sporadic Sharp Wave” response category for the “Sharp Waves” item was rarely used by the evaluators, and the “Paroxysmal Activity” item had a binary trend with only two responses used by the evaluators (“0 = None” and “1 = Paroxysmal Activity/spikes”). The model fit indices were AIC 3424.02 and BIC 3508.21.

To optimize the model, we excluded the “Background Frequency” item and collapsed the response categories for “Sharp Waves” and “Paroxysmal Activity.” This change resulted in a more favorable model with improved fit indices (AIC 2494.16 and BIC 2554.29). The results of the optimized model are presented in Table 3, which shows the ${\text{sqNPS}}_{i,\text{age},\text{evaluator}}$ loadings for each GTE item. Notably, all items loaded well onto the latent factor, with “Focal Abnormality” and “Paroxysmal Activity” indicating the highest values at 0.98 and 0.73, respectively.

**Table 3:** Item response theory results: factor loadings for the latent neurophysiological status (${\text{sqNPS}}_{i,\text{age},\text{evaluator}}$).

Grand total EEG items	Loadings
Diffuse slow activity	0.65
Paroxysmal activity	0.73
Focal abnormality	0.98
Sharp waves	0.56

**Figure 2:**
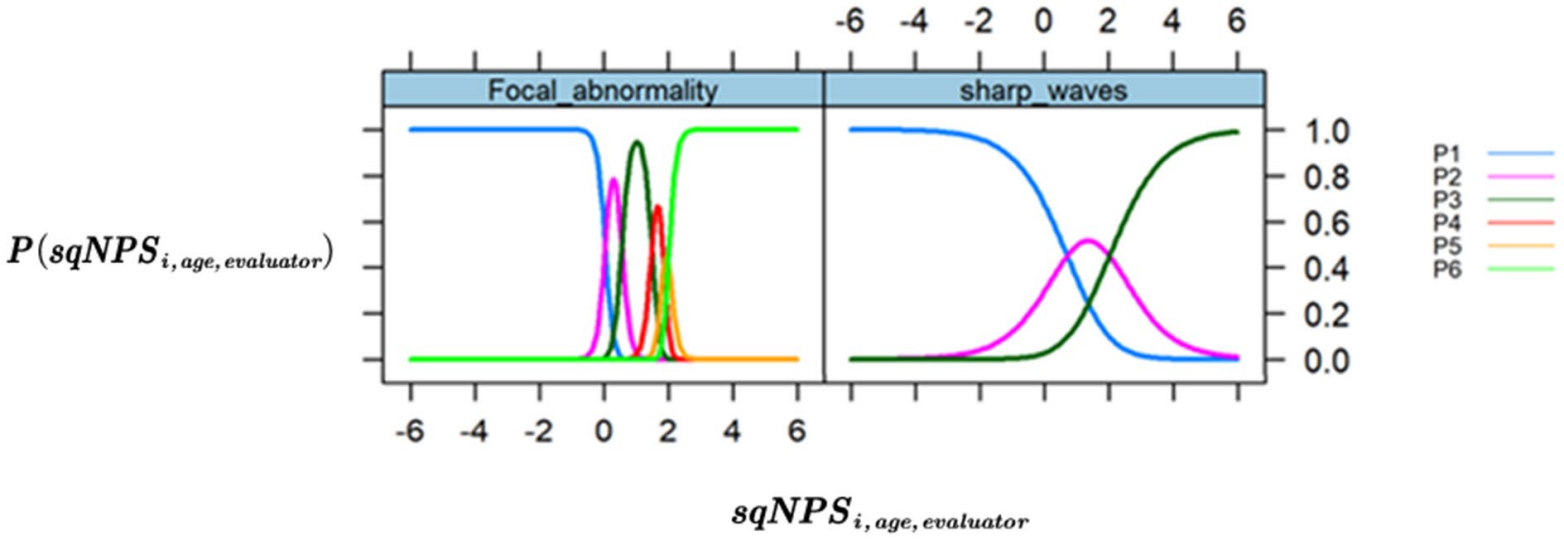
Trace/ICC plots. The *x*-axis is the value of the latent variable ${\text{sqNPS}}_{i,\text{age},\text{evaluator}}$ (neurophysiological status), and the y-axis is the $P\left({\text{sqNPS}}_{i,\text{age},\text{evaluator}}\right)$ that shows the probability or chance of occurrence for each response category across different levels of NPS. (Left) “Focal Abnormality,” the item with the highest loading (0.976). (Right) “Sharp waves,” the item with the lowest loading (0.563).

Figure 2 shows the trace plots for the items with the largest and smallest loadings, showing a curve for every response category under the item and representing it against the latent factor ${\text{sqNPS}}_{i,\text{age},\text{evaluator}}$. The trace plots for the optimized model show that all categories of response items are well represented.

### 3.4. Univariate LME model comparing nutritional groups

The statistical model used to assess group differences is as follows:


$$\begin{array}{l} {\text{sqNPS}}_{i,\text{age},\text{evaluator}}=1+\underset{\text{Fixed Effect}}{\underset{︸}{\text{group}+\text{age}+\text{group}:\text{age}}}\\ \;\;\;\;\;\;\;\;\;\;\;\;\;\;\;\;\;\;\;\;\;\;\;\;\;\;\;\;\;\;\;\;\;\;\;+\underset{\text{Covariate}}{\underset{︸}{\text{sex}+\text{ecology}}}+\underset{\text{Random Effect}}{\underset{︸}{\text{Subjects}/\text{Evaluator}}} \end{array}$$

We compared two models (1) that included random effects for subjects only and (2) random effects for both subjects and evaluators. However, the ANOVA results indicated no significant difference between the model that only considered participant-based random effects and the other model that included participants plus evaluator effects (*p* = 1). Consequently, we proceeded with the simpler model that only included participant effects. Additionally, the interaction between Group and Age was not significant (*p* = 0.81), suggesting that the nutritional group differences remain constant over the lifespan (slopes are parallel).

As presented in Table 4, the fixed effect of the nutrition group was highly significant (*p* < 0.001), indicating a strong association between malnutrition history and the outcome variable, ${\text{sqNPS}}_{i,\text{age},\text{evaluator}}$. Age was also found to be a significant predictor of ${\text{sqNPS}}_{i,\text{age},\text{evaluator}}$(*p* < 0.001), while Sex and Standard of Living (Ecology factor) did not demonstrate any significant association with ${\text{sqNPS}}_{i,\text{age},\text{evaluator}}.$ It is worth noting that our analyses showed that ${\text{sqNPS}}_{i,\text{age},\text{evaluator}}$ has c ≥ 1, which is an index of local sensitivity to non-ignorability (ISNI) of missingness pattern. These results indicate that the missingness pattern of the EEG data was ignorable, and that the missing data was not significantly different from the observed data. Therefore, the results are reliable and can be generalized to the population.

**Table 4:** Linear Mixed effect results: Estimates with Missing at Random (MAR) assumption, the second column is *p*-values and 95% lower, and upper confidence interval (CI) and last two columns are Sensitivity analysis estimates for the violation of MAR assumption.

	Estimate	Value of *p*	95% Lower CI	95% Upper CI	ISNI	*c*
(Intercept)	0.08	0.46	−0.14	0.31	0.157	0.63
Nutrition Group (PEM)	−0.69	**<0.001**	−0.97	−0.40	−0.025	4.98
Age	0.01	**0.01**	0.00	0.01	−0.004	0.74
Sex (Male)	0.16	0.08	−0.02	0.34	0.004	17.61
Ecology Factor	−0.02	0.67	−0.11	0.07	0.002	22.97
Age: Nutrition Group (PEM)	0.00	0.81	−0.01	0.01	0.001	5.67

Significance (*p* < 0.05) is shown in bold.

### 3.5. Association of sqNPS with MoCA factor

We initially conducted a comparison between the Total MoCA scores of two groups (PEM, CON) where the former had a mean score of 24.74 (±4.23) and the latter had a mean score of 28.04 (± 3.16). The results showed a significant difference between the two groups, with higher scores in the control group indicating less cognitive impairment. To validate the Neurophysiological status latent score (${\text{sqNPS}}_{i,\text{age},\text{evaluator}}$) generated from semi-quantitative EEG (sqEEG), we compared our results with factor MoCA.

To assess if ${\text{sqNPS}}_{i,\text{age},\text{evaluator}}$ is comparable with the MoCA, we applied a polytomous IRT model for dimensionality reduction of the seven MoCA subscales. The resulting latent MoCA factor ($MF_{i,\text{age}=45-51\text{yrs}}$) was found to have high factor loadings on language (0.92), attention (0.89), and abstraction (0.81). We investigated the association between the $MF_{i,\text{age}=45-51\text{yrs}}$ and ${\text{sqNPS}}_{i,\text{age},\text{evaluator}}$ scores at two-time points separately, as the longitudinal analysis was not possible due to MoCA being measured only in adult life. Table 5 shows the results of the associational analysis.

**Table 5:** Standardized *β*-estimates showing associations between sqNPS at 5–11 years and 45–51 years with MF scores for PEM and CON groups, combined (*N* = 202)—linear regression analyses adjusted for sex, age, and standard of living.

	Age = 5–11 years				Age = 45–51 years			
	*β*	Value of *p*	95% Lower CI	95% Upper CI	*β*	Value of *p*	95% Lower CI	95% Upper CI
(Intercept)	0.38	0.76	−1.47	2.23	−0.65	0.35	−2.12	0.82
${\text{sqNPS}}_{i,\text{age},\text{evaluator}}$	−0.23	0.02	−0.43	−0.03	−0.22	0.00	−0.36	−0.07
Ecology	−0.20	0.04	−0.39	−0.01	0.01	0.83	−0.13	0.16
Sex (Male)	−0.18	0.35	−0.55	0.19	−0.02	0.90	−0.32	0.28
Age	0.30	0.75	−1.59	2.20	0.71	0.33	−0.72	2.14

As shown in Table 5, the ${\text{sqNPS}}_{i,\text{age},\text{evaluator}}$ scores were significantly associated with the MoCA Factor (MF) at both time points, even after adjusting for standard of living, sex, and age. Notably, there was a significant association between ${\text{sqNPS}}_{i,\text{age}=5-11\text{yrs},\text{evaluator}}$ measured between the ages of 5–11 years and $MF_{i,\text{age}=45-51\text{yrs}}$ scores assessed 40 years later. This association was statistically significant (*p* = 0.02), indicating that early EEG abnormalities associated with malnutrition predict adult cognitive impairment. Interestingly, this association was also observed in middle adulthood with ${\text{sqNPS}}_{i,\text{age}=45-51\text{yrs},\text{evaluator}}$(*p* = 0.003), indicating that the relationship between childhood ${\text{sqNPS}}_{i,\text{age},\text{evaluator}}$ values and cognitive impairment persists over the lifespan.

### 3.6. Discriminant analysis for prediction power

We applied the stable sparse classifier (SSC) to predict biomarkers for two different time points: childhood and adulthood. As a prediction method, the classifier was not designed to handle longitudinal data. SSC was employed under three different settings to compare the prediction accuracy of both methods independently and when combined. The implemented models are

-
${\text{sqNPS}}_{i,\text{age},\text{evaluator}}$

-
$z_{i,\text{age}}\left(e,\omega \right)$

- a joint model for ${\text{sqNPS}}_{i,\text{age},\text{evaluator}}$ and $z_{i,\text{age}}\left(e,\omega \right)$

Table 6 presents the performance of the sparse stable classifier in classifying childhood malnutrition-based on $z_{i,\text{age}}\left(e,\omega \right)$ and ${\text{sqNPS}}_{i,\text{age},\text{evaluator}}$ and their combination, both at the ages of 5–11 years and 45–51 years. The mean area under the receiver operating characteristic (ROC) curve for 1,000 cross-validations and the standard deviation is reported for each classification scenario. The results indicate that the sparse stable classifier achieved a high level of accuracy for ages 5–11 years, with mean AUC values ranging from 0.83 ± 0.007 [$z_{i,\text{age}=5-11\text{yrs}}\left(e,\omega \right)$] to 0.84 ± 0.007 (${\text{sqNPS}}_{i,\text{age}=5-11\text{yrs},\text{evaluator}}$). Notably, the combination of $z_{i,\text{age}=5-11\text{yrs}}\left(e,\omega \right)$ and ${\text{sqNPS}}_{i,\text{age}=5-11\text{yrs},\text{evaluator}}$ yielded the highest mean AUC value of 0.92 ± 0.005, indicating that integrating these two measures can improve the accuracy of childhood malnutrition classification.

**Table 6:** Performance of the sparse stable classifier: the mean area under the ROC curve for 1,000 cross-validations and standard deviation.

Age	$z_{i,\text{age}}\left(e,\omega \right)$	${\text{sqNPS}}_{i,\text{age},\text{evaluator}}$	Both
5–11 years	0.83 ± 0.007	0.84 ± 0.007	0.92 ± 0.005
45–51 years	0.82 ± 0.008	0.72 ± 0.01	0.87 ± 0.007

The classification was performed separately at both time points. Columns show different implementation settings with (1) spectral quantitative EEG $(z_{i,\text{age}}\left(e,\omega \right)$), (2) Neurophysiological status (${\text{sqNPS}}_{i,\text{age},\text{evaluator}}$), and (3) a combination model using both $z_{i,\text{age}}\left(e,\omega \right)$ and ${\text{sqNPS}}_{i,\text{age},\text{evaluator}}$.

In contrast, the performance of the sparse stable classifier in middle adulthood (for ages 45–51 years) was slightly lower, with mean AUC values ranging from 0.72 ± 0.01 (${\text{sqNPS}}_{i,\text{age}=45-51\text{yrs},\text{evaluator}}$) to 0.82 ± 0.008 ($z_{i,\text{age}=45-51\text{yrs}}\left(e,\omega \right)$). However, combining both modalities outperformed the individual methods (mean AUC of 0.87 ± 0.007).

### 3.7. Multivariate linear mixed effect model

The SSC identified a set of stable biomarkers from $z_{i,\text{age}}\left(e,\omega \right)$, which were utilized to construct a latent variable via factor analysis known as quantitative NPS (${\text{qNPS}}_{i,\text{age}}$). On the other hand, we created an average score over evaluators for ${\text{sqNPS}}_{i,\text{age},\text{evaluator}}$ resulting in ${\text{sqNPS}}_{i,\text{age},⊙}$.


$$\begin{array}{l} \left[{\text{qNPS}}_{i,\text{age}},{\text{sqNPS}}_{i,\text{age},}\right]=1+\underset{\text{Fixed Effect}}{\underset{︸}{\text{group}+\text{age}+\text{group}:\text{age}}}\\ \;\;\;\;\;\;\;\;\;\;\;\;\;\;\;\;\;\;\;\;\;\;\;\;\;\;\;\;\;\;\;\;\;\;\;\;\;\;\;\;\;+\underset{\text{Covariate}}{\underset{︸}{\text{sex}+\text{ecology}}}\;+\underset{\text{Random Effect}}{\underset{︸}{\text{Subjects}}} \end{array}$$

The main MLME model is given above; three different variations of this model were compared where

- Model 1: $\left[{\text{qNPS}}_{i,\text{age}},0\right],$ and
- Model 2: $\left[0,{\text{sqNPS}}_{i,\text{age},}\right],$
- Model 3: $\;\left[{\text{qNPS}}_{i,\text{age}},{\text{sqNPS}}_{i,\text{age},}\right].$

M1 and M2 were compared with model 3 with the log-likelihood test. The null hypothesis is that the models have no significant difference in performance. Table 7 shows the model comparison results, where the log-likelihood measures how well a model fits the data, while the log-likelihood ratio (L Ratio) compares the improvement in model fit between two nested models.

**Table 7:** ANOVA for linear mixed effect models based on the comparison between the performance of quantitative neurophysiological status (${\text{qNPS}}_{i,\text{age}}$), semi-quantitative NPS (${\text{sqNPS}}_{i,\text{age},⊙}$), and a combination model using both [${\text{qNPS}}_{i,\text{age}}$,$\;{\text{sqNPS}}_{i,\text{age},}$].

MLME models	Log-likelihood	Test	L ratio	*p*-value
$\left[{\text{qNPS}}_{i,\text{age}},0\right]$	−506.91	${\text{qNPS}}_{i,\text{age}}$ vs. Both	54.381	<0.0001
$\left[0,{\text{sqNPS}}_{i,\text{age},}\right]$	−489.3	${\text{sqNPS}}_{i,\text{age},}$ vs. Both	19.3	0.02
$\left[{\text{qNPS}}_{i,\text{age}},{\text{sqNPS}}_{i,\text{age},}\right]$	−479.7	-	-	-

The first column shows the multivariate linear mixed effect (MLME) models, followed by log-likelihood for each model. The last three columns pertain to the comparative test between models. These columns are the test performed, the log-likelihood ratio (L Ratio), and the corresponding value of *p*s for each test.

Table 7 shows that the joint model $\left[{\text{qNPS}}_{i,\text{age}},{\text{sqNPS}}_{i,\text{age},⊙}\right]$ had the highest log likelihood (−479.7), indicating the best overall fit to the data. In contrast, the $\left[{\text{qNPS}}_{i,\text{age}},0\right]$ model had the lowest log-likelihood (−506.91). The difference in performance between the $\left[{\text{qNPS}}_{i,\text{age}},0\right]$ model and $\left[{\text{qNPS}}_{i,\text{age}},{\text{sqNPS}}_{i,\text{age},⊙}\right]$ model was statistically significant (L Ratio = 54.381, *p* < 0.0001). This suggests that adding ${\text{sqNPS}}_{i,\text{age}}$ improves the model’s accuracy.

## 4. Discussion

Our findings indicate noticeable distinctions between protein-energy malnutrition (PEM) and the control (CON) groups. Specifically, we observed a higher frequency of abnormal Grand Total EEG (GTE) item scores in the PEM group compared to control during both childhood and middle adulthood. The Supplementary material presents the raw scores of the GTE items for each group and time point, including the mean and standard deviations as evaluated by two independent evaluators for each study. The histograms illustrate the graphical trends between groups and conditions, with the control group consistently displaying lower scores over time, except for the focal abnormality item. We employed the semi-quantitative EEG (sqEEG) method to generate Neurophysiological Status (${\text{sqNPS}}_{i,\text{age},\text{evaluator}}$) latent scores from GTE items to ensure an unbiased assessment.

Our sqEEG-based approach utilized Item Response Theory (IRT) to generate latent ${\text{sqNPS}}_{i,\text{age},\text{evaluator}}$. It is important to note that our IRT-based approach differs from the Taboada-crispi et al. (2018) study on the same cohort in several aspects. Firstly, our study included a larger dataset and more time points, providing a more comprehensive and reliable analysis. Additionally, we excluded non-reliable items from the data and also modified and collapsed the response categories which were not informative. This resulted in an optimized IRT model with better fit indices. This allowed us to create standardized scores comparable across evaluators, which provided valuable insights into the neurophysiological status (${\text{sqNPS}}_{i,\text{age},\text{evaluator}}$) of the PEM and control groups.

Among the GTE items, two significant contributors to the IRT-based latent neurophysiological status were “Focal abnormalities (0.98)” and “paroxysmal activity (0.73).” Focal abnormalities are characterized by an increase in bilateral and multifocal abnormalities in the EEG recordings, which contradicts the expected normal maturation process of the brain (Segalowitz et al., 2010). On the other hand, Paroxysmal activity indicates Interictal Epileptic Discharge (IED), as noted by Kural et al. (2020) and Tatum and Shellhaas (2020). According to a meta-analysis encompassing data from over 50,000 subjects by Shelley et al. (2008), paroxysmal activity prevalence in normal children ranges from 0.8 to 18.6%, and in adults, it ranges from 0.3 to 12.3%. In childhood, PEM individuals have higher rates of paroxysmal activity (21.4% vs. 14.3% in controls). The paroxysmal activity in controls is well within the range as reported by Shelley et al. (2008); however, some other studies report lower activity values for healthy children (Darwish et al., 2017; Redka et al., 2020). In adulthood, the paroxysmal activity in the BNS cohort was 18.6% for the PEM group vs. 14.4% in controls, with PEM values exceeding the reported normative range and with slightly elevated values for the CON group (Shelley et al., 2008). The focal abnormalities observed bilaterally and multifocally in the recordings and higher paroxysmal activity may result from early adversity, as previously reported that early environmental adversity can lead to altered brain networks (Wijeakumar et al., 2019).

The standardized IRT scores based on two evaluators at each time point were analyzed using a Linear Mixed Effect (LME) model, which revealed no significant differences when incorporating evaluators as random effects. The additional random effects did not improve the model’s performance, thus indicating the absence of evaluator bias. The LME results revealed highly significant differences between the groups (*p* < 0.001). The sensitivity index for missingness yielded robust results, which aligns with our previous findings (Bosch-Bayard et al., 2022). Furthermore, ${\text{sqNPS}}_{i,\text{age},\text{evaluator}}$ showed a significant association with age (*p* = 0.01). However, the interaction between Group and Age was insignificant (*p* = 0.81), suggesting that the observed differences in nutritional groups remained consistent across the lifespan, with no reversal of the effects of malnutrition as subjects matured. The non-significant interaction term between age and nutritional group indicates agreement between the two studies.

Linear mixed-effects results showed no significant association between ${\text{sqNPS}}_{i,\text{age},\text{evaluator}}$ and Sex. While numerous studies have demonstrated that sex can influence brain anatomy and function, EEG studies do not consistently observe this association (Matsuura et al., 1985; Bosch-Bayard et al., 2001, 2022; Li et al., 2022). On the other hand, despite a more disadvantaged socioeconomic status in the PEM group, sqNPS showed no significant relationship with ecology. A previous study conducted by Harmony et al. (1988) determined that socioeconomic status played an important role in predicting deviant spectral-qEEG results. However, some factors may explain the discrepancies between our findings in the BNS cohort and that study. Firstly, during the 1977–1978 study, Barbados did not experience the same extremes of socioeconomic conditions as other underserved populations. Additionally, the PEM children in the study were involved in a 12-year government intervention program, potentially mitigating any disparities in outcomes due to disadvantaged home environments. This finding is consistent with previous research from the BNS cohort (Galler et al., 1983; Bosch-Bayard et al., 2022). While socioeconomic status is crucial, it may not fully encompass the extent of adversity being addressed here (Farah, 2017).

We compared our method with MoCa to validate our findings based on ${\text{sqNPS}}_{i,\text{age},\text{evaluator}}$. We found that semi-quantitative Neurophysiological Status in childhood and middle adulthood was significantly associated with cognitive outcomes on the MoCA. Increased EEG abnormalities in the PEM group at ages 5–11 years predicted elevated cognitive decline 40 years later and over the life span. These findings signal the lasting neurological and neurobehavioral consequences of early childhood malnutrition and extend our earlier observations of cognitive deficits in the BNS cohort (Galler et al., 1983; Waber et al., 2011, 2014). We have previously reported associations between the spectral-qEEG and MoCA outcomes (Razzaq et al., 2020; Razzaq, 2021; Bosch-Bayard et al., 2022).

The comparison between Neurophysiological State (${\text{sqNPS}}_{i,\text{age},\text{evaluator}}$) with spectral quantitative EEG [spectral-qEEG: $z_{i,\text{age}}\left(e,\omega \right)$] was in two steps (1) stable sparse classifier (SSC) and (2) Log Likelihood of Multivariate linear mixed-effects (MLME) models. The SSC analysis showed that the combination of $z_{i,\text{age}}\left(e,\omega \right)$ and ${\text{sqNPS}}_{i,\text{age},\text{evaluator}}$ yielded the highest predictive power, with an average AUC value of 0.92 ± 0.005. Additionally, we compared three MLME models to identify the best biomarker for longitudinal effects, and the joint MLME model $\left[{\text{qNPS}}_{i,\text{age}},{\text{sqNPS}}_{i,\text{age},⊙}\right]$ had the highest log-likelihood (−479.7). Notably, the qEEG only model $\left[{\text{qNPS}}_{i,\text{age}},0\right]$ has the lowest log-likelihood. Our findings indicate that while ${\text{sqNPS}}_{i,\text{age}}$ and ${\text{qNPS}}_{i,\text{age}}$ produce similar results, ${\text{sqNPS}}_{i,\text{age},⊙}$ offers unique information that cannot be obtained solely through ${\text{qNPS}}_{i,\text{age}}$. Combining ${\text{sqNPS}}_{i,\text{age},⊙}$ with ${\text{qNPS}}_{i,\text{age}}$ proves to be more effective than using either method independently. Therefore, adopting a multimodal approach may enhance the efficacy of future studies in this field.

Our study provides valuable insights into the distinct neurophysiological patterns observed in individuals with PEM compared to the control group. Our findings highlight the importance of considering a history of childhood malnutrition when analyzing EEG recordings and GTE scores in underserved populations. This study further supports earlier reports showing that EEG changes resulting from early childhood malnutrition predict accelerated cognitive decline at ages 45–51 years (Bosch-Bayard et al., 2022) and adds to the literature on the long-term brain and behavioral consequences of early malnutrition. Findings from the Dutch famine study are focused on the effects of famine exposure during gestation on cognitive decline (De Groot et al., 2011; Wiegersma et al., 2022) and brain aging in adulthood using structural and functional fMRI (Franke et al., 2018; Boots et al., 2022). Two long-term studies of survivors of the Chinese Great Leap Forward Famine have reported on early cognitive decline in adults exposed to undernutrition during gestation and late childhood (Wang et al., 2016) and following famine exposure in early childhood (1–3 yrs. of age) (Kang et al., 2017). However, neither famine study included neuroimaging assessments during early life. Furthermore, famine studies have generally relied on reports of period of famine exposure but have not directly evaluated the nutritional status and health of the child at the time of exposure. Unlike these earlier studies, we examined all children during their malnutrition episode and throughout childhood and have incorporated neuroimaging measures, specifically the EEG, in both children and adults.

In summary, this study contributes to the existing literature by examining the impact of early malnutrition on the brain using EEG measures and relating these to cognitive decline in adulthood. The sqEEG thus represents another cost-effective measure that can be used to identify individuals at risk for malnutrition effects on brain development in resource-poor settings. With the development of qEEG, the sqEEG analysis is less represented in the scientific literature. Even if spectral quantitative EEG has been employed repeatedly and successfully to demonstrate differences between groups in many settings, we recognize that it does not consider other cross spectra, as in Li et al. (2022), or nonlinear and nonstationary features which provide rich information about the underlying electrophysiological changes. Note that conventional EEG analysis is based solely on the visual examination of the continuous tracings, which may be highly subjective and, therefore, a disadvantage. Nevertheless, neurophysiologists can provide unique and valuable information about EEG resting state activity which can be partially quantified using sqEEG.

## 5. Limitations and future work

This study has several limitations which may have impacted the findings. One limitation is the attrition of the original sample of participants with EEG in 1978 (*N* = 258). However, the composite sample employed for this analysis (*N* = 149) represents 58% of the total cohort, which is substantial in comparison with other international longitudinal studies (Pollitt et al., 1993; Victora et al., 2008; Gustavson et al., 2012; Launes et al., 2014; Sánchez and Escobal, 2020). Further, this is one of the few studies that has followed individuals exposed to a limited period of postnatal malnutrition during the first year of life who were then fully rehabilitated following their illness. The sensitivity procedure we employed to test the influence of missing data in the analysis ruled out any potential effect of attrition on the results.

A second limitation pertains to potential environmental influences that may not have been documented. Even though the healthy controls were selected from the same classrooms as the PEM group, the nutrition groups nonetheless differed with respect to their childhood and adult standard of living (Galler and Ramsey, 1985; Galler et al., 2012). Although we adjusted for these effects in all our statistical models, it is possible that we failed to include all environmental factors and childhood adversities that may have contributed to their impaired cognitive and neurological outcomes. Other examples of childhood adversity that were more likely to be present in the PEM group and may have played a role in these findings include child maltreatment (Hock et al., 2017) and maternal depression (Salt et al., 1988; Galler et al., 2010; Waber et al., 2011). It is well known that childhood adversity plays a major role in brain and behavioral development (Fox et al., 2010; Teicher et al., 2016). Future EEG studies will address these additional childhood adversities more comprehensively.

Finally, in further developing our work using sqEEG, we would like to carry out sqEEG with a more comprehensive EEG grapho-element ontology, such as SCORE (Beniczky et al., 2017). When comparing sqEEG with qEEG, we will augment the qEEG biomarker set, as mentioned before, with functional connectivity measures and nonlinear features. Finally, we also plan to examine a broader set of behavioral and cognitive outcomes that are available in the BNS archival records.

## 6. Conclusion

This research affirms the long-term impact of early childhood malnutrition on brain function, as assessed through semiquantitative EEG (sqEEG) and spectral quantitative EEG (qEEG). This study found that increased sqEEG abnormalities during childhood predicted an accelerated cognitive decline that persisted for over five decades into middle adulthood. Both the MoCA and qEEG results corroborated these findings. Overall, the study highlights the distinct features of both sqEEG and spectral-qEEG and underscores the value of utilizing a combined approach to develop an effective predictive biomarker for malnutrition-related cognitive impairment in children who are at high risk. It also highlights the need for an augmented qEEG measure set, including functional connectivity and nonlinear/nonstationary features.

## Data availability statement

The raw data supporting the conclusions of this article will be made available by the authors, without undue reservation.

## Ethics statement

The studies involving human participants were reviewed and approved by Informed consent was provided by parents of all participants when they were initially enrolled in the study under Protocol E1962, approved by the Boston University Medical Center Institutional Review Board and the Ethics Committee of the Barbados Ministry of Health. Current oversight (in adulthood) is provided by the Massachusetts General Brigham IRB (Protocol No. 2015P000329), the Cuban Neurosciences Center (2017/02/17/CNEURO), and the Barbados Ministry of Health. All adult participants provided written informed consent and were compensated for their time and travel expenses. Written informed consent to participate in this study was provided by the participants’ legal guardian/next of kin.

## Author contributions

PV-S, MB-V, and JG conceived, organized, and executed this project in collaboration. JG contributed as co-founder and director of the 50+ year Barbados Nutrition Study. AC-R, CS-M, AR, JG, CB, and SA were responsible for data acquisition. AC-R, TV-A, YG, QT, VB, and AR undertook the data curation and scoring of the GTE. PV-S, IM, LG-G, MB-V, FR, and JG developed the methods. FR undertook the statistical analyses, with the participation of AC-R and JB-B. FR, UR, AR, JG, MB-V, and PV-S prepared the manuscript. MB-V, PV-S, and JG acquired the funding. All authors contributed to the article and approved the submitted version.

## Footnotes

^1^ https://data.unicef.org/topic/nutrition/malnutrition/

^2^ www.ifcn.info

^3^ https://globalbrainconsortium.org/project-norms
